# Methodological Approach of the Iron and Muscular Damage: Female Metabolism and Menstrual Cycle during Exercise Project (IronFEMME Study)

**DOI:** 10.3390/ijerph18020735

**Published:** 2021-01-16

**Authors:** Ana B. Peinado, Victor M. Alfaro-Magallanes, Nuria Romero-Parra, Laura Barba-Moreno, Beatriz Rael, Cristina Maestre-Cascales, Miguel A. Rojo-Tirado, Eliane A. Castro, Pedro J. Benito, Carmen P. Ortega-Santos, Elena Santiago, Javier Butragueño, Antonio García-de-Alcaraz, Jesús J. Rojo, Francisco J. Calderón, Alberto García-Bataller, Rocío Cupeiro

**Affiliations:** 1 LFE Research Group, Faculty of Physical Activity and Sport Sciences, Universidad Politécnica de Madrid, 28040 Madrid, Spain; vm.alfaro@upm.es (V.M.A.-M.); n.romero@upm.es (N.R.-P.); laura.barba@upm.es (L.B.-M.); b.rael@alumnos.upm.es (B.R.); cristina.maestre@upm.es (C.M.-C.); ma.rojo@upm.es (M.A.R.-T.); elianeaparecidacastro@gmail.com (E.A.C.); pedroj.benito@upm.es (P.J.B.); javier.butragueno@gmail.com (J.B.); antoniogadealse@gmail.com (A.G.-d.-A.); jesusjavier.rojo@upm.es (J.J.R.); franciscojavier.calderon@upm.es (F.J.C.); rocio.cupeiro@upm.es (R.C.); 2 Department of Health and Human Performance, Faculty of Physical Activity and Sport Sciences, Universidad Politécnica de Madrid, 28040 Madrid, Spain; 3 Department of Sports Sciences and Physical Conditioning, Faculty of Education, Universidad Católica de la Santísima Concepción, 2850 Concepción, Chile; 4 College of Health Solutions, Arizona State University, Phoenix, AZ 85004, USA; carmenpatriciaortega@gmail.com; 5 Clínica Tambre, 28002 Madrid, Spain; elen_sant@hotmail.com; 6 Faculty of Educational Sciences, Universidad de Almería, 04120 Almería, Spain; 7 Department of Sports, Faculty of Physical Activity and Sport Sciences, Universidad Politécnica de Madrid, 28040 Madrid, Spain; alberto.garcia@upm.es

**Keywords:** hormones, iron metabolism disorders, hepcidin, endurance training, resistance training, creatine kinase, follicular phase, luteal phase, women

## Abstract

**Abstract:**

*Background*: The increase in exercise levels in the last few years among professional and recreational female athletes has led to an increased scientific interest about sports health and performance in the female athlete population. The purpose of the IronFEMME Study described in this protocol article is to determine the influence of different hormonal profiles on iron metabolism in response to endurance exercise, and the main markers of muscle damage in response to resistance exercise; both in eumenorrheic, oral contraceptive (OC) users and postmenopausal well-trained women. *Methods*: This project is an observational controlled randomized counterbalanced study. One hundered and four (104) active and healthy women were selected to participate in the IronFEMME Study, 57 of which were eumenorrheic, 31 OC users and 16 postmenopausal. The project consisted of two sections carried out at the same time: iron metabolism (study I) and muscle damage (study II). For the study I, the exercise protocol consisted of an interval running test (eight bouts of 3 min at 85% of the maximal aerobic speed), whereas the study II protocol was an eccentric-based resistance exercise protocol (10 sets of 10 repetitions of plate-loaded barbell parallel back squats at 60% of their one repetition maximum (1RM) with 2 min of recovery between sets). In both studies, eumenorrheic participants were evaluated at three specific moments of the menstrual cycle: early-follicular phase, late-follicular phase and mid-luteal phase; OC users performed the trial at two moments: withdrawal phase and active pill phase. Lastly, postmenopausal women were only tested once, since their hormonal status does not fluctuate. The three-step method was used to verify the menstrual cycle phase: calendar counting, blood test confirmation, and urine-based ovulation kits. Blood samples were obtained to measure sex hormones, iron metabolism parameters, and muscle damage related markers. *Discussion*: IronFEMME Study has been designed to increase the knowledge regarding the influence of sex hormones on some aspects of the exercise-related female physiology. Iron metabolism and exercise-induced muscle damage will be studied considering the different reproductive status present throughout well-trained females’ lifespan.

**Trial registration:**

The study was registered at Clinicaltrials.gov NCT04458662 on 2 July 2020.

## 1. Background

Female endogenous hormonal fluctuations during the menstrual cycle or exogenous hormones from an oral contraceptive cycle may have potential effects on exercise performance [1,2,3]. The female reproductive system involves numerous hormonal and regulatory components, affecting not only the reproductive system, but also with an impact on female metabolism, thereby influencing exercise performance [1]. Thus, it is important know how the hypothalamic-pituitary-ovarian axis works and affects other systems and metabolism related to exercise, in order to adapt and optimise training sessions and competitions. 

The menstrual cycle is a sequence of circamensal rhythms domineered by the feedback loops within the hypothalamus-pituitary-ovarian axis [4]. The average menstrual cycle length is 28 days, with an interindividual variation of 21–35 days in healthy adult women [5], and it involves repetitive cycles of follicle development, ovulation, and preparation of the endometrium for possible implantation of an embryo [6]. This process begins in the hypothalamus, with the pulsatile secretion of gonadotropin-releasing hormone (GnRH). It travels through the pituitary portal venous system to the anterior pituitary gland [6], stimulating the synthesis and pulsatile secretion into circulation of luteinizing hormone (LH) and follicle-stimulating hormone (FSH). These two gonadotropins targets the ovaries, where stimulates the secretion of sex steroid hormones 17β-estradiol and progesterone [6,7].

Oral contraceptives are traditionally used for birth control or cycle control purposes as they eliminate the explained circamensal changes in female sex hormones [8]. The exogenous hormones provided by oral contraceptives trigger negative feedback on the hypothalamus-pituitary axis that inhibits the gonadotropin surge [9,10]. As a result, downregulation of the endogenous steroid hormones estrogen and progesterone takes place, and ovulation is avoided [9,10]. In addition, from the mid-40 s, a gradual process of reproductive senescence called climacteric begins, resulting in a significant decline in the ovary’s contribution to plasma estradiol and progesterone [11,12]. Consequently, menstrual irregularity occurs, with periods of amenorrhea lasting over 60 days and up to 12 months. This events ultimately leads to a physiological state called menopause and characterized by a permanent drop in estradiol and progesterone levels and the loss of the menstrual cycle [12].

The literature is conflicting so far about the secondary physiological effects of oestrogen and progesterone, specifically on iron metabolism [13,14,15,16,17] and muscle damage [18], most likely due to the large changes in these hormones during the menstrual cycle or hormone administration [19]. Many of these studies have been conducted in animals under hormone treatment and in most cases supplying or injecting much higher hormonal doses than the endogenous or exogenous hormones from a natural menstrual or oral contraceptive cycle [19]. In addition, most studies have not considered these changes over an exercise stimulus making it difficult to extrapolate data to trained premenopausal women. For this reason, it is important to study the whole-body function and the natural sexual hormone fluctuations as an integral system to contemplate the different stimulus affecting physiology complex and exercise performance. 

Iron is an essential element with many important roles in the body, since it is required for haemoglobin synthesis the transport of oxygen throughout the body [20]. Iron deficiency is one of the most common nutritional deficiencies worldwide, especially affecting premenopausal women due to the additional iron demands of menstruation and pregnancy [21,22]. In addition, athletes are frequently diagnosed with inadequate iron levels, particularly those involved in endurance sports [23,24,25]. Regular training practices, on occasion accompanied by inadequate iron intake, may compromise iron status through intravascular haemolysis, increased erythropoietic demands, gastrointestinal bleeding or sweat iron loss [26], which could lead to iron deficiency or, even worse, iron deficiency anaemia. Therefore, the prevention of iron deficiency, especially in females participating in endurance-type exercise, is a major concern as this condition, even in the context of iron deficiency without anemia, presents several health disturbances [27]. 

Resistance training is widely used by female athletes to improve performance and it is also popular due to its health-related benefits, i.e., increased strength and lean body mass [18]. In fact, other chronic conditions such as osteoporosis or sarcopenia may be improved or delayed by resistance training [28,29]. However, this exercise modality could involve repeated eccentric contractions or strenuous muscle activities that may elicit muscle damage and inflammation [30]. In this regard, the ovarian hormones have been reported to be connected with skeletal muscle, which is the largest tissue containing oestrogen receptors [31,32]. Oestrogens may play a protective role due to their membrane-stabilising characteristics and hence oestrogens may prevent exercise-induced muscle damage (EIMD) and subsequent inflammation [30]. Nevertheless, the literature supporting this protective role of oestrogens is inconclusive due to the small number of studies considering women in different stages of their lifespan or intra-subject studies including different menstrual cycle phases or contraceptive phases [18].

Thus, the main objective of the project described here is to determine the influence of different hormonal profiles on iron metabolism in response to endurance exercise, and the main markers of muscle damage and inflammation in response to resistance exercise, in eumenorrheic, oral contraceptive (OC) users and well-trained postmenopausal women. As secondary objectives, we propose to study the influence of different hormonal environments in body composition, cardiorespiratory variables and strength training measurements. Moreover, we also aim to analyse the potential influence of several genetic variants over the physiological variables measured in this project, as well as their allelic distribution within an active female sample.

## 2. Methods/Design

### 2.1. Study Design

This project was an observational controlled randomised counterbalance study performed in physically active and healthy women. The project consisted of two sections carried out at the same time: iron metabolism (study I) and muscle damage (study II). Three different groups participated in each study: eumenorrheic, OC users and well-trained postmenopausal women. These study groups performed an exercise protocol in a randomised and counterbalanced order at different moments according to their sex hormonal profile. For study I, the exercise protocol consisted of an interval running test, whereas the study II protocol was based on a resistance exercise trial. In both studies, eumenorrheic participants were evaluated at three specific moments of the menstrual cycle, i.e., early follicular phase (EFP), late follicular phase (LFP) and mid-luteal phase (MLP); OC users performed the trial at two moments: withdrawal phase (WP) and active pill phase (APP). Finally, postmenopausal women were tested only once, since their hormonal status does not fluctuate. All of them received dietary recommendations and recorded their training sessions during their participation period.

### 2.2. Participants

**Figure 1:**
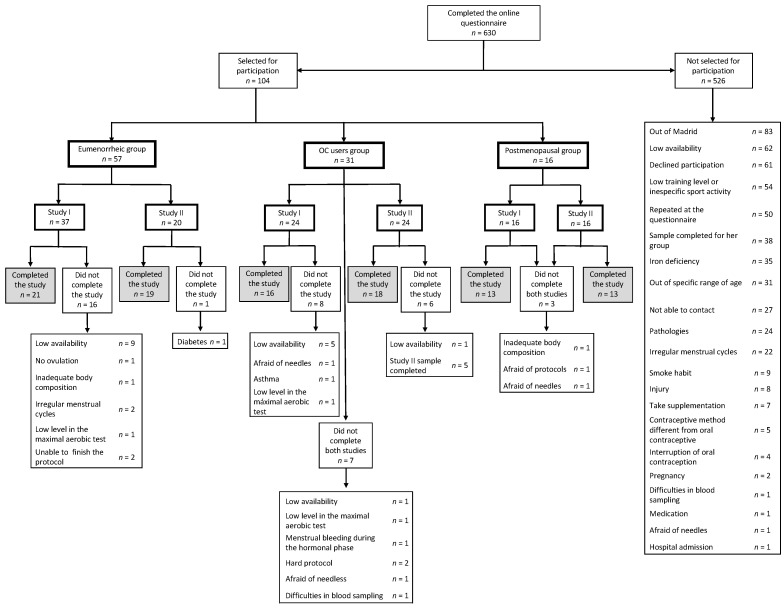
Consort flow diagram. Eumenorrheic women selected to participate in study I and II were different, whereas OC users and postmenopausal women were recruited to participate in both studies. OC: Oral contraceptive.

One hundred and four (104) physically active and healthy women were selected to participate in the IronFEMME Study, 57 of whom were eumenorrheic, 31 were OC users and 16 were postmenopausal women. Regarding study I, 37 eumenorrheic, 31 OC users and 16 postmenopausal women were recruited for the study. Regarding study II, 20 eumenorrheic, 31 OC users and 16 postmenopausal women were recruited for the study. Table 1 shows the descriptive data of the women selected to participate in the study. Eumenorrheic women selected to participate in study I and II were different, whereas OC users and postmenopausal women were recruited to participate in both studies. Figure 1 shows the flow diagram of the participants in the study. 

### 2.3. Inclusion and Exclusion Criteria

All inclusion and exclusion criteria were previously determined through an online participant screening questionnaire. Participants were required to meet the following inclusion criteria: (a) healthy adult females between 18 and 40 years old for the eumenorrheic and OC user groups or under 60 years old for postmenopausal women; (b) not presenting iron deficiency anaemia (serum ferritin >20 μg/L, haemoglobin >115 μg/L and transferrin saturation >16%); (c) performing endurance training between 3 and 12 h per week (study I); or (d) experienced in resistance training performing at least 30 min session two times per week during a minimum of a year (study II). The exclusion criteria were: (a) irregular menstrual cycles; (b) any existing disease and/or metabolic or hormonal disorder; (c) any musculoskeletal injury in the last six months prior to the beginning of the project; (d) any surgery interventions (e.g., ovariectomy) or other medical conditions that would be exacerbated by the exercise protocols; (e) regular use of medication or dietary supplements that could affect the results (e.g., nonsteroidal anti-inflammatory drugs); (f) taking medication that alters vascular function (e.g., tricyclic antidepressants, α-blockers, β-blockers, etc.); (g) pregnancies in the year preceding; (h) inadequate body composition; and (i) smoking. 

A eumenorrheic cycle, defined as normally occurring menstrual cycles from 24 to 35 days in length [5], was required during the six months previous to the study for the eumenorrheic group. The OC users had to have consumed monophasic contraception during the six previous months before the beginning of the study. The daily APP dose of ethinylestradiol varied with the different brands from 20 μg to 35 μg; and the progestogen dosage varied as follows: 100 μg of levonorgestrel, 250 μg of norgestimate, 2000 μg of dienogest or 3000 μg of drosperinone. Postmenopausal was defined according to the Stages of Reproductive Aging Workshop (STRAW) criteria [33], as amenorrhea for ≥12 months excluding oophorectomy [12].

### 2.4. Recruitment

Subjects were recruited by using a diversity of advertisements published in social media, regional sports competitions and federations. The applicants were filtered through an initial questionnaire previously fulfilled, containing information about nutrition, health, menstrual cycle and training. The voluntary subjects who met the inclusion criteria were called by phone to confirm these data and others regarding menstrual cycle. After that, a study ID was assigned to each participant depending on the study group (study I or study II) and their hormonal condition (eumenorrheic, OC users or postmenopausal), which was included in the database. Moreover, a written consent form and an information report about the IronFEMME Study were sent by email to the participants. Finally, they were asked to notice the first day of their next menstrual bleeding, in order to start the screening protocol. 

### 2.5. Sample Size Estimation

Sample size was determined using GPower software, version 2 (Heinrich-Heine-University, Düseldorf, Germany). For this calculation, the previous results of the study Sim et al. [34] were used, as the hepcidin response was evaluated throughout a monophasic oral contraceptive cycle (WP and APP). There are no studies that measure the main variable of the study I (hepcidin) in the different hormonal phases of a eumenorrheic menstrual cycle. Taking these data as reference, the power analysis suggested an *n* = 21 to produce a statistical power of 0.80 with a significance level of *p* < 0.05. A drop-out rate of 20% was applied, so an n of 25 participants per group was the calculated sample size. Simple size determination for study II was done considering creatine kinase (CK) as the main variable of this study. The results published by Sipaviciene et al. [35] were used to calculate the sample size. They showed the CK response 24 h after exercise in follicular phase (1200 ± 800 IU/L) and luteal phase (750 ± 250 IU/L) of a sample of eumenorrheic women (*n* = 18). The power analysis suggested an *n* = 14 to produce a statistical power of 0.80 with a significance level of *p* < 0.05. A drop-out rate of 20% was applied, so an *n* of 18 participants per group was the estimated sample size.

### 2.6. Randomization

Participants were randomised individually by a researcher external to the project who had no contact with the participants prior to or during the trial. This researcher had no intellectual or personal investment in the study design or outcomes. The stratified random sampling method was used to determine randomly and counterbalanced allocation, and the order of the menstrual cycle phase by which the participants would begin each study (i.e., study I and II). Additionally, due to the difficulty of finding OC users and postmenopausal women, these study groups participated in both studies (i.e., study I and II). The order in which these women participated in each study was also randomised. In this way, the learning effect of the exercise protocols was distributed homogeneously, avoiding its influence.

In the eumenorrheic group, the order in which the tests were carried out was randomised according to the phases of the menstrual cycle, using the following codes: 1-EFP; 2-LFP; 3-MLP. In this way, the following test orders were randomised: 1-2-3; 2-3-1; 3-1-2; 2-1-3 and 1-3-2. For the OC users group, the phases of the oral contraceptive cycle received the following codes: 1-WP and 2-APP. Similarly, these codes were also randomised: 1-2 and 2-1. Lastly, since the postmenopausal group only performed one single test, no randomisation was needed, except the order of participation in each study.

### 2.7. Menstrual Cycle Monitoring and Phase Determination 

In the present study, different phases throughout the menstrual cycle were selected for the different study groups. The phases for the eumenorrheic group were EFP, LFP and MLP, whereas those for the OC users were WP and APP. 

**Figure 2:**
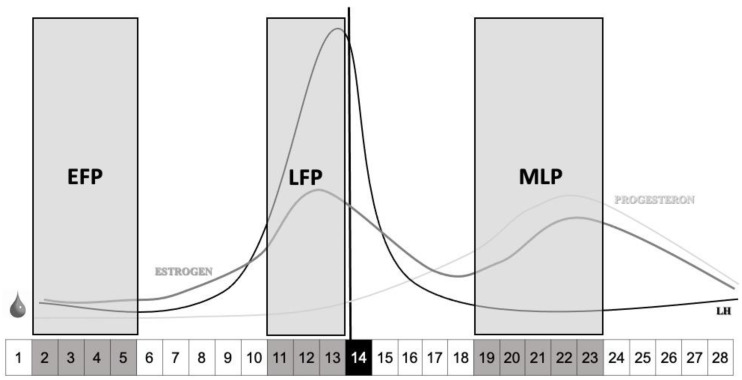
Timeline of testing across a regular menstrual cycle of 28 days. The testing day for the early-follicular phase (EFP) were between the day 2 to 5 of the cycle; between the days 11 to 13 for the late-follicular phase (LFP) considering day 14 as ovulation; and for the mid-luteal phase between days 19 to 23 of the cycle. LH: Luteinizing hormone.

Considering the first day of the cycle the onset of menstruation for the eumenorrheic group, the days of testing were: between the 2nd and the 5th day of the cycle for the EFP, between one and three days before the ovulation day for the LFP, and between five and nine days following ovulation for the MLP (Figure 2). These specific phases were selected in order to analyse different hormonal environments as suggested by the literature [36]: low sex hormonal levels in the EFP, low progesterone but high oestrogen levels in the LFP and elevated both progesterone and oestrogen levels in the MLP. In order to ensure this, we applied three different methods (see below): calendar-based counting, urinary LH measurement and serum hormone analysis.

The OC users mapped their cycle based on their pill packaging, starting the first day of the cycle with the first inactive pill or WP. For this phase, participants performed the test between days 4–7, then they attended between the second or third week of the consumption phase (days 15–28) to perform the other test during the APP. For this group, the specific days for the different phases were selected in order to observe the following hormonal concentrations: higher endogenous hormone concentrations due to more elevated ovarian activity for the WP, and a constant circulation of exogenous hormone concentrations due to the accumulation of active pills for the APP. If participants reported missing two or more consecutive pills in one cycle, testing was delayed until the next cycle. In order to verify that pill consumption had been carried out correctly, serum hormone analysis was performed at each phase. It is important to observe higher oestrogen levels during the late WP compared to the APP due to the ovarian activity produced by pill withdrawal during those days. However, progesterone levels should remain stable and low in both phases.

#### 2.7.1. Calendar-Based Counting

The first method to identify phases of the menstrual cycle was the calendar-based counting derived from the traditional Ogino method. This indirect method sets the self-reported onset of menses as day 1, and the phases are then established by counting days from this point [36]. More specifically and before that, participants were asked to record information about the length of their last six menstrual cycles (number of days from the cycle onset to the next one). This blinded data was provided to a gynaecologist, who confirmed that menstrual cycles were regular and estimated the ovulation day. Over the average of the last menstrual cycle’s length, the ovulation day was established as the middle day of that average period (e.g., if the obtained average length of those months is 28, the ovulation day was set as day 14 of the cycle). Once the ovulation day was estimated for each participant, then the menstrual cycle phases were calculated in order to set the testing days in each phase. The EFP starts with menstrual bleeding and it usually lasts between 4–7 days, depending on the participant. All the participants of this study were tested between days 2 and 5 for the EFP, in order to obtain the lowest sex hormones concentrations. To estimate the timeframe selected for the LFP, we took as a reference the estimated ovulation day by the gynaecologist, and the exercise protocol was scheduled between one and three days before ovulation day. Finally, the MLP was estimated according to Schaumberg et al. [37], who stated that this begins between 20–22 days following the onset of menstruation. These authors determined the highest likelihood of correct MLP classification according to an optimal progesterone concentration. These criteria were used with those participants having cycles between 28 and 30 days. For those ones with shorter (24–27 days) or longer (31–35 days) cycles we applied the same criteria but adjusting the phases to the corresponding ovulation day. However, the main limitation of this method is that it does not distinguish between ovulatory and anovulatory or luteal phase-deficient (progesterone limit <16 nmol/L) cycles; some authors have stated that luteal phase-deficient and anovulation often occur in active women with regular bleeding [36,37]. Therefore, to accurately identify the phases of the menstrual cycle, we combined this method with urinary LH measurements and serum hormone analysis.

#### 2.7.2. Urinary LH Measurement

A home urine-based predictor kit (Ellatest, Alicante, Spain) was used to identify the LH surge and subsequent ovulation. Our participants collected their mid-morning urine (always at the same time of day) from three to five days before the scheduled LFP testing day until the test result was positive. If any participant did not get a positive test result, then they waited until the next cycle. If, after three menstrual cycles, the participant did not obtain a positive test result, then she was excluded from the study for having anovulatory cycles. If the test was positive, ovulation would occur within 14–26 h after the urinary peak (in most cases) [38]. The test was performed by inserting the test strip into the urine according to the manufacturer’s instructions and then waiting approximately 10 min to see a positive or negative result for urinary LH. The LFP testing day was correct when it had been carried out 0–2 days before the positive test result. In addition, this method allowed us to estimate the MLP more precisely since, according to the literature, an optimal luteal phase should occur between 7–9 days after a positive ovulation prediction test [37]. Nevertheless, although the use of an LH surge ovulation prediction kit may increase the likelihood of accurately estimating the point of ovulation and the timing of testing in the MLP, some authors report that this does not exclude luteal phase-deficient cycles (up to 30% of participants who experience a positive urinary ovulation test) [37,39]. Therefore, additional menstrual cycle phase verification was applied in this study in order to fully confirm the different phases of the menstrual cycle. 

#### 2.7.3. Serum Hormone Analysis

The measurement of serum hormone concentrations is a direct method and considered the gold standard for research purposes. 17β-estradiol, progesterone, LH and FSH were measured before testing in each of the menstrual cycle phases selected for the study. This method requires the collection of a venous blood sample (approximately 8 mL). The blood sample is then left to clot before centrifugation. The serum is separated and stored frozen at −80 °C. Later, sex hormones were analysed by the Spanish National Centre of Sport Medicine (Madrid, Spain). A rise in progesterone from the follicular phase to the luteal phase was used to verify that ovulation had occurred [36]. The majority of the studies in the literature had set a minimum progesterone limit of 16 nmol/L required as a reliable indicator of an ovulatory non-luteal phase-deficient cycle [2,40,41]. In addition, this method was also important to measure oestrogen concentrations and to confirm the LFP with a high oestrogen concentration. This phase presents a characteristic hormonal environment with low progesterone concentrations and high oestrogen levels. In addition, it is important to observe higher oestrogen concentrations during this phase than during the luteal phase and higher progesterone concentrations compared to the EFP, but lower than 6.36 nmol/L [42]. If any participant did not meet the minimum progesterone levels required for the MLP or the oestrogen concentrations were significantly low with regard to the criteria mentioned above, the participant was asked to repeat the test at that specific phase of the menstrual cycle or were asked to drop out of the study.

### 2.8. Diet and Exercise Restrictions and Recommendations

All participants were instructed to avoid pro-inflammatory food 48 h prior to testing, testing day and 24 h after testing, including the breakfast 48 h after testing (e.g., red meat, processed meat, salty snacks, cold meats or plant-based alternatives [43,44,45,46]). They were instructed to eat one of the options from the standardised diet types as follows: Breakfast: scrambled eggs with spinach and a piece of fruit or three pieces of fruit with 50 g of dried fruit (e.g., walnuts) and wholegrain (>70% of the product must have whole grains) toast with one teaspoon of tahini or peanut butter; Lunch/dinner: 100% whole grain cereals (e.g., barley) or potato/sweat potato baked, or legumes, with poultry, plant-based protein (e.g., tofu), fish or seafood, with a mix of a cooked and raw vegetables; cooking oils were limited to extra virgin olive oil or extra virgin avocado oil; Beverages: water; Snacks: pistachios with citrus fruit (no juices were allowed) or a tuna sandwich with wholegrain bread or raisins and nuts. We collected two different types of digital food records. First, to assess the dietary intake the day before and day after testing days, we collected a 72-h dietary recall. Second, we asked all participants to record a weekly three-day food dietary food record form (two weekdays and one weekend day) during the intervention. All the food record forms required the participant to register the date, time of the day, size, quantity and description of the food and beverages consumed. All participants were provided with instructions and an Excel file to record all dietary intakes. 

### 2.9. Ethical Issues

The protocols and procedures of the IronFEMME Study were in approved by the Ethical Principles for Medical Research Involving Human Subjects of the World Medical Association Declaration of Helsinki (1964) and further amendments. Before participating in this research, all subjects were carefully informed about the possible risks and benefits of the project, being required to read and sign an institutionally approved consent form. The IronFEMME Study was approved by the Human Research Ethics Committee of the Universidad Politécnica de Madrid. Access to the database was restricted to the researchers that participated in the IronFEMME Study. Therefore, the data and information obtained in the project was considered as confidential following current Spanish legislation regulating personal data protection (Organic Law 3/2018). 

### 2.10. Study Interventions

#### 2.10.1. Screening Protocol

On the first day, our volunteers came to our laboratory between 8:00 a.m. and 10:00 a.m. in a rested and fasted state. It was during the EFP for the eumenorrheic group, during the WP for the OC users group, and at any time for the postmenopausal group. Firstly, they signed all the consent forms and participant’s weight and height were recorded. Then, blood pressure was measured to check that participants did not suffer from hypertension and thus avoid possible risks related to blood pressure during exercise [47]. Baseline blood samples were collected for a complete blood count, genetic testing, biochemistry and hormonal analyses. After the blood sample was collected, absorptiometry by dual-energy X-ray (DXA) was done. This screening session was completed with a maximal aerobic test (study I) or a strength assessment of the lower limbs through a one repetition maximum (1RM) test for the parallel back-squat exercise using a plate-loaded barbell (study II). 

#### 2.10.2. Blood Count and Hormonal Measurements

Fasting blood samples were collected via a brachial arterial catheter for a complete blood count and biochemistry in order to check inclusion criteria and the participant’s health. Additionally, 17β-estradiol, progesterone, prolactin, LH, FSH and thyroid-stimulating hormone (TSH) were measured in order to control and verify the normal sexual function of the eumenorrheic, OC users and postmenopausal groups. 

#### 2.10.3. Dual Energy X-Ray Absorptiometry (DXA)

A baseline DXA analysis was performed on each of the participants. We assured that the participants wore the least possible clothing to be accurate. In addition, they did not carry any metal objects. After that, participants were laid down facing upwards within the area marked by the machine for analyses performing, according to the manufacturer’s instructions. Then, the test was carried out, using a GE Lunar Prodigy apparatus (GE Healthcare, Madison, WI, USA), and scan analyses were performed using GE Encore 2002 software v. 6.10.029 (GE Healthcare, Madison, WI, USA). During the 7–10 min of the analysis carried out by the apparatus, the participants were asked to move as little as possible. To do this, they were helped by binding their legs at the ankles with tape. The variables measured were fat mass, fat-free mass, bone mineral content and bone mineral density.

#### 2.10.4. Study I: Endurance Protocol

##### Maximal Aerobic Test

On the same day as the screening protocol, after a meal and rest (a minimum of 2 h after feeding), participants performed an incremental running exercise to exhaustion on a computerised treadmill (H/P/COSMOS 3PW 4.0, H/P/Cosmos Sports & Medical, Nussdorf-Traunstein, Germany) to determine their peak oxygen uptake (VO_2peak_). Expired gases were measured breath-by-breath with a Jaeger Oxycon Pro gas analyser (Erich Jaeger, Viasys Healthcare, Friedberg, Germany), the validity and reliability of which have been previously demonstrated [48,49]. Heart rate was continuously monitored with a 12-lead ECG. Participants began with a warm-up of 3 min at 6 km/h. Once the warm-up finished, the speed was set at 8 km/h and then increased by 0.2 km/h every 12 s until exhaustion. A slope of 1% was set throughout the test to simulate air resistance. The recovery phase included an active recovery of 2 min (walking at a speed of 6 km/h) and a passive recovery of 3 min (sitting on a chair). Volunteers did not perform physical activity, consume caffeine or any take supplements 24 h prior to the test. This test was carried out in the EFP (between days 2 and 5 of the menstrual cycle) for the eumenorrheic group [5] and in the WP (between days 4 and 7) for the OC users group. 

To verify that VO_2peak_ was reached, a confirmatory test was carried out as suggested in previous studies [50,51] after a 5 min recovery period after the maximal aerobic test [51]. The test consisted of a 3-min warm-up (2 min at 50% of the maximal velocity reached in the maximal aerobic test and 1 min at 70% of the same velocity) [50]. After the warm-up, velocity was set at 110% of the maximal velocity reached in the maximal aerobic test. Participants ran at this velocity until exhaustion [52]. If participants did not run for at least 1 min at this velocity, the confirmatory test was not taken into account for VO_2peak_ measurement and it was determined only with the maximal aerobic test. Lastly, participants performed a recovery test of 2 min at 6 km/h.

VO_2peak_ was determined as the mean of the three highest VO_2_ measurements in the maximal aerobic test [53]. This value was considered if it was not less than 3% compared to the value obtained in the confirmatory trial. If the value was less than 3%, VO_2peak_ was calculated as the mean of the three highest VO_2_ values recorded during the last 30 s of the confirmatory trial. The maximal aerobic speed (vVO_2peak_) was recorded as the minimum speed required to elicit VO_2peak_ [54]. If the VO_2peak_ was determined by the confirmatory trial, a linear regression was used to calculate the corresponding velocity. Then, the speed equivalent to 85% of the vVO_2peak_ was calculated for use in the interval running protocol. The main variables measured were oxygen uptake (VO_2_), pulmonary ventilation (VE), carbon dioxide production (VCO2), respiratory exchange ratio (RER) and heart rate (HR).

##### Testing Procedure Day

**Figure 3:**
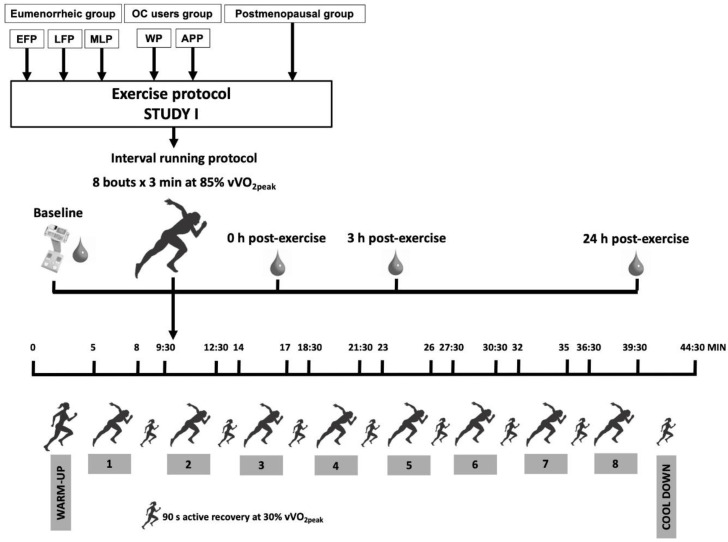
Protocol of the testing procedure day of study I. OC: Oral contraceptive; EFP: Early-follicular phase; LFP: Late-follicular phase; MLP: Mid-luteal phase; WP: Withdrawal phase; APP: Active pill phase; vVO_2peak_: Maximal aerobic speed.

Participants came to the laboratory abstaining from alcohol, caffeine and any moderate/intense physical activity or sport practice the day before. Protocols started between 8:00 and 10:00 a.m. in the morning to avoid diurnal variability of hepcidin [55] and having breakfast at least 2 h earlier. In addition, participants replicated the same breakfast in each protocol performed in the different menstrual cycle phases and followed the nutritional recommendations. Body composition measurement by bioelectrical impedance and blood sample collection were done just before the running protocol. Subsequently participants started the interval running protocol. Figure 3 shows the tests performed during the testing procedure day of study I.

##### Baseline Measurements 

Firstly, participants performed a bioelectrical impedance test. We ensured that participants fulfilled the requirements to carry out electrical biofeedback [56]. After that, they were placed on the bioelectrical impedance machine, with their feet placed on the marked area and holding the grips with their hands, with extended arms placed near the body. Then, the test was carried out, using a hand to foot bioelectrical impedance analyser TANITA BC-418 (Tanita Corp., Tokyo, Japan). The variables measured in the bioelectrical impedance test were fat mass, fat-free mass, water and impedance. In addition, blood pressure was measured before the exercise protocol.

Blood samples were collected at baseline from the antecubital vein to analyse the following parameters related to iron homeostasis and inflammation: iron, ferritin, transferrin, C reactive protein (CRP), interleukin-6 (IL-6), tumour necrosis factor α (TNF-α) and hepcidin. Additionally, concentrations of 17β-estradiol, progesterone, prolactin, LH, FSH and TSH were analysed to confirm the menstrual cycle phase in which participants performed each interval running protocol. 

##### Interval Running Protocol

After baseline measurements, participants performed an interval running protocol. This consisted of a 5-min warm-up at 60% of the vVO_2peak_ followed by eight bouts of 3 min at 85% of the vVO2_peak_ with 90 s recovery at 30% of the vVO_2peak_ between bouts. Finally, a 5-min cool down was performed at 30% of the vVO_2peak_. This protocol was previously reported by Sim et al. [57] to stimulate a hepcidin response at 3 h post-exercise. During exercise, VO_2_, VCO_2_, HR, VE and RER ventilatory variables were continuously measured using the same apparatus as mentioned for the maximal aerobic test. Additionally, rate of perceived exertion (RPE) and perceived readiness (PR) were respectively measured by RPE Borg 6–20 scale [58] and PR Nurmekivi 1–5 scale [59]. Participants were asked regarding their RPE in the last 5 s of the warm-up, and during every running bout and cool down. The PR scale was applied in the last 5 s of the warm-up, recoveries 1 to 7 and cool down. 

##### Post-Testing Measurements

Immediately after the protocol, a blood sample was collected. Additionally, 3 h and 24 h after finishing the interval running protocol, blood samples were collected. The same iron homeostasis and inflammation variables were analysed as at baseline. 

#### 2.10.5. Study II: Resistance Protocol

##### RM Estimation 

On the same day of the screening protocol, the 1RM in the parallel back-squat exercise was determined by using the Powerlift App (Carlos Balsalobre-Fernández, Madrid, Spain) [60], based on the force (load)-velocity relationship [61]. Participants performed a 5-min cycle-ergometer warm-up and some mobility and dynamic stretching exercises. After that, the test consisted of 4 sets of 1 repetition with submaximal loads proportionally increased between 70% and 90% of participants’ maximum self-reported. To record the videos, a researcher (always the same) held an iPhone 6S (Apple Inc., Cupertino, CA, USA) in portrait position and recorded each lift with a high-speed camera (240 Hz), from the right side of the participant, in order to see the full range of motion as close as possible. The beginning of the lift was considered the first frame in which the barbell went up (thighs parallel to the floor), and the end of the lift was consider the first frame in which the barbell ended its vertical displacement (hips and knees extended). Since this procedure required manual selection by the researcher, two independent observers analysed the same video. High inter-observer reliability (ICC) has been reported in previous validation studies (ICC > 0.9) [62]. 

##### Testing Procedure Day

**Figure 4:**
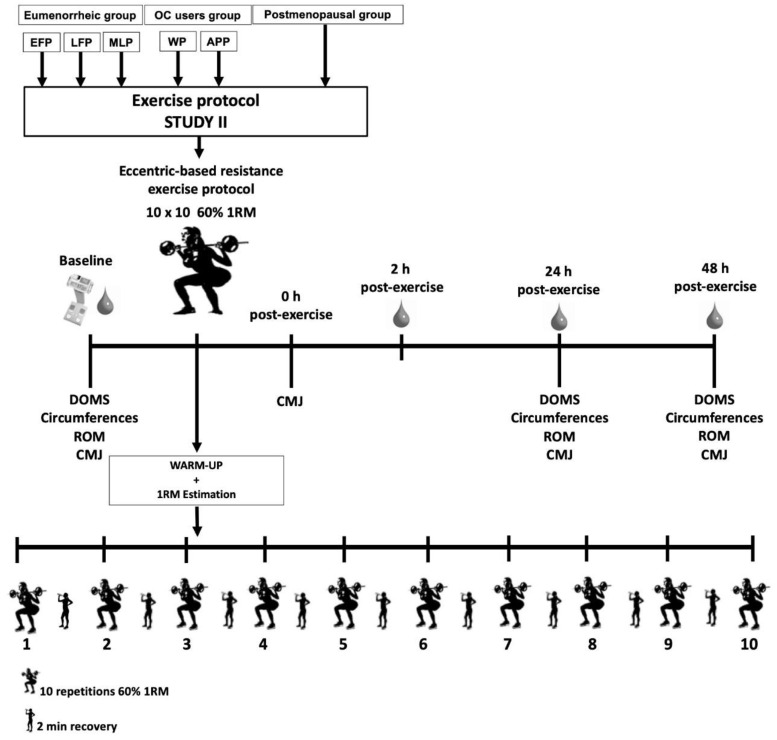
Protocol of the testing procedure day of study II. OC: Oral contraceptive; EFP: Early-follicular phase; LFP: Late-follicular phase; MLP: Mid-luteal phase; WP: Withdrawal phase; APP: Active pill phase; DOMS: Delayed onset muscle soreness; ROM: Range of movement, CMJ: Counter movement jump test.

Participants were asked to refrain from any physical activity and abstain from alcohol and caffeine for 48 h prior to testing. During these sessions, an eccentric-based resistance exercise protocol was performed consisted of 10 sets of 10 repetitions of plate-loaded parallel back squats at 60% of their previously calculated 1RM. This protocol was previously reported by Macdonald et al. [63] to produce substantial EIMD. Perceived delayed onset muscle soreness (DOMS), thigh and calf circumference, hip and knee range of movement (ROM), and counter movement jump test (CMJ) performance, were assessed prior to exercise, 24 h and 48 h post-exercise. Additionally, CMJ was assessed immediately post-exercise. Serum blood samples were obtained at baseline, 2 h, 24 h and 48 h post-exercise. Figure 4 describes the measurements performed during the testing procedure day of study II.

##### Baseline Measurements

Firstly, bioelectrical impedance and blood pressure tests were performed as previously mentioned in the baseline measurements of the endurance protocol of study I. In addition, blood samples were collected from the antecubital vein at baseline in order to analyse the following markers related to muscle damage and inflammation: CK, myoglobin (Mb), lactate dehydrogenase (LDH), TNF-α, CRP and IL-6. The concentrations of 17β-estradiol, progesterone, prolactin, LH, FSH and TSH were also analysed to confirm participants performed the test during the appropriate menstrual cycle phase.

##### Delayed Onset Muscle Soreness

Muscle soreness were measured using a Visual Analogue Scale (VAS) [64,65,66]. Participants were requested to rate the level of soreness in thighs and glutes experienced during a parallel-unweighted squat from 0 mm (no pain at all) to 100 mm (unbearable pain). The VAS has been reported to be a valid and reliable measure of DOMS (interclass correlation > 0.96) [64,65,66].

##### Circumferences

A standard centimetre-marked tape was used in order to measure changes in muscle girth as an indirect marker of oedema. Midthigh and midcalf limb girths were assessed on the right side of the body according to the International Society for the Advancement of Kinanthropometry (ISAK) guidelines. Midthigh girth is defined as the halfway point between the anterior superior iliac spine and the proximal aspect of the patella [63], and was measured with the subject standing erect horizontally around the point previously described. Midcalf girth was set at the maximal segmental girth [67]. All landmark sites were marked with permanent marker and remained marked for each phase testing sessions to ensure reliability across trials.

##### Range of Movement

Hip and knee passive ROM were measured using a manual goniometer accurate to 1° (Jamar 360° steel goniometer, Jamar, Greendale, WI, USA) after completion of 5-min cycle-ergometer warm-up. For hip passive ROM, each participant laid prone on the floor whilst a researcher flexed her right hip (both knees extended) until the point of discomfort. A decrease in the angle of the hip indicated an increase in mobility. Then, to measure knee ROM, participants performed a modified kneeling lunge with left leg with the trunk in an upright position, placing their left knee in line with their left ankle and aligning their lower left leg perpendicular to the floor so that the right hip was stretched to the point of discomfort [63]. This hip angle was also registered and reproduced in subsequent occasions. After positioning, researcher passively flexed the right knee until reaching the point of discomfort. A decrease in the angle of the knee indicated an increase in mobility. 

##### Counter Movement Jump Test

After completion of the ROM assessment, participants performed a CMJ, which was measured by using My Jump v.5.0.6 iOS App (Carlos Balsalobre-Fernández, Madrid, Spain) [68]. To record the video, the researcher held the iPhone 6S used before to measure 1RM, facing the participants and zoomed in on their feet. Participants were instructed to maximise jump height and were given verbal encouragement. The flight time was calculated by identifying take-off (first frame in which feet did not touch the floor, with full knee and ankle extension) and landing (first frame in which feet touched the floor) on the video and jump height was obtained. As mentioned earlier, high-speed videos (240 Hz) were recorded and processed by the same researcher.

##### Eccentric-Based Resistance Exercise Protocol

After completion the above assessments, some mobility and dynamic stretching exercises and a more specific squat-based warm-up was performed with moderate loads: two sets of five repetitions at 50% and 60% of their 1RM calculated in the screening session. After that, the 1RM was estimated again in each eccentric session by performing a quick test with the Powerlift App based on the full test previously performed in the screening session. Once the 1RM test was completed, the 1RM load was provided by the app from the force-velocity profile.

The EIMD protocol consisted of 10 sets of 10 repetitions of plate-loaded barbell parallel back squats, with 2 min of recovery between sets. The weight was set at 60% of their 1RM weight. Squats were performed at a tempo of 4 s eccentric movement, 1 s pause at the bottom, 1 s concentric movement and 1 s pause at the top of the lift to focus on the eccentric phase of the exercise for greater muscle damage [63]. The tempo was controlled using an interval timer, with the investigator signalling the subject regarding the changes in the lifting phase. Perceived exertion from every set was also obtained by using a 10-point scale from 0 (extremely easy) to 10 (extremely hard) [69]. 

##### Post-Testing Measurement

As mentioned above, CMJ was assessed immediately post-exercise and, 2 h after the eccentric-based protocol, a blood sample was collected. Additionally, 24 h and 48 h post-exercise, DOMS, thigh and calf circumference, hip and knee ROM, and CMJ performance were assessed; blood samples were also collected. The same muscle damage and inflammation variables were analysed as at baseline.

### 2.11. Blood Sampling Collection and Analysis

All venous blood samples were obtained using a 21-gauge (0.8 × 19 mm, Terumo^®^, Shibuya, Japan) needle. Blood samples for complete blood counts were collected in a 3 mL K3E EDTA K3 tubes (Vacuette^®^, Greiner Bio-One GmbH, Kremsmünster, Austria) and immediately sent for analysis to the clinical laboratory of the Spanish National Centre of Sport Medicine (Madrid, Spain) for analysis. Blood samples for serum variables were collected in a 9 mL Z serum separator clot activator tube (Vacuette^®^, Greiner Bio-One GmbH, Kremsmünster, Austria). Following inversion and clotting (60 min at room temperature), the whole blood was centrifuged (LMC-3000 version V.5AD, Biosan, Riga, Latvia) for 10 min at 3000 rpm to obtain the serum (supernatant). After that, serum was pipetted into 600 µL aliquots, transferred into Eppendorf tubes and stored frozen at −80 °C until further analysis. The serum samples were delivered to the previous laboratory in order to analyse all blood parameters. Samples were allowed to defrost at room temperature and were then homogenised on a vortex.

Total 17β-estradiol, progesterone, FSH, LH, prolactin, TSH and IL-6 were measured with a COBAS E411 (Roche Diagnostics GmbH, Mannheim, Germany), using electrochemiluminescence immunoassay (ECLIA) technology. Inter- and intra-assay coefficients of variation (CV) reported by the laboratory for each variable were, respectively: 11.9% and 8.5% at 93.3 pg/mL and 6.8% and 4.7% at 166 pg/mL for 17β-estradiol; 23.1% and 11.8% at 0.7 ng/mL and 5.2% and 2.5% at 9.48 ng/mL for progesterone; 5.3% and 1.8% at 1.2 mIU/mL for FSH; 5.2% and 1.8% at 0.54 mIU/mL for LH; 5.0% and 4.0% at 300 mIU/L for prolactin; 4.6% and 1.5% at 3.82 µIU/mL for TSH; 8.5% and 6.0% at 17.3 pg/mL for IL-6.

Serum iron was analysed by spectrophotometry. Ferritin, transferrin, CRP, CK, Mb, and LDH were analysed by turbidimetry. Colorimetry and turbidimetry were conducted using an AU400 clinical analyser (Beckman Coulter, Brea, CA, USA) and Beckman reagents. TNF-a was measured using a chemiluminescent enzymatic immunoassay (IMMULITE 1000 system; Siemens Healthineers AG, Munich, Germany). The reaction was calibrated following the manufacturer’s instructions, and controls were measured after calibration and subsequently in each analysis batch. Inter- and intra-assay CV were, respectively: 1.77% and 0.66% at 28.34 µg/dL and 1.23% and 0.65% at 105.54 µg/dL for iron; 3.71% and 2.24% at 25 ng/mL for ferritin; 0.86% and 0.64% at 284 mg/dL for transferrin; 6.4% and 4.3% at 0.21 mg/L for CRP; 3.2% and 1.0 at 270 U/L for CK; 4.2% and 2.9% at 45.2 µg/L for Mb; 1.5% and 1.1% at 157 U/L for LDH; 6.5% at 17 pg/mL and 3.5% at 34 pg/mL for TNF-a.

Duplicate serum samples were sent to the Department of Laboratory Medicine at Radboud University Medical Centre (Hepcidinanalysis.com, Nijmegen, The Netherlands) for the measurement of hepcidin-25 serum concentrations. Serum hepcidin was measured by a combination of weak cation exchange chromatography and time-of-flight mass spectrometry (WCX-TOF MS) using a stable hepcidin-25+40 isotope and secondary reference material [70] as the internal standard for quantification [71]. Peptide spectra were generated on a Microflex LT matrix-enhanced laser desorption/ionisation TOF MS platform (Bruker Daltonics, Fremont, CA, USA). Hepcidin-25 concentrations were expressed as nmol/L (nM). The lower limit of quantification of this method was 0.5 nM. Inter-assay CV for hepcidin was 4.6% at the 11.0 nM level and 8.3% at the 2.7 nM level. Reference values can be found at http://www.hepcidinanalysis.com/provided-service/reference-values (accessed on 22 April 2020). All values were determined using secondary reference material for hepcidin assays, which value is assigned by a primary reference material, allowing traceability to the internationally recognised Système International [70]. 

### 2.12. Genetic Testing 

To investigate the possible influence of the genetic profile on physiological responses to the testing protocols, we have selected different genetic single nucleotide polymorphisms (SNPs) potentially related to the variables analysed in the study. For the selection of SNP, we took into account their prevalence in the general population and/or their physiological impact and/or function. The potential roles for all of the selected variants were documented, and they were located in genes coding for proteins influencing the expression of the hepcidin gene (TMPRSS6, encoding for Matriptase-2) [72,73,74], iron metabolism and its regulation (HFE and TF) [72,75,76,77,78], proteins used as post-exercise muscle damage markers or involved in the response of exercise-induced muscle damage (ACTN3, CKMM, and MLCK) [79,80,81,82,83,84], or proteins related to the inflammatory response (IL6 and TNF) [85,86,87,88,89,90].

We assessed the prevalence and distribution of these polymorphisms in the different population groups recruited (eumenorrheic, oral contraceptive or postmenopausal), and analysed associations within the studied variables and response to the testing protocols.

Apart from general informed consent, all subjects signed a specific informed consent form that allows sample storage and genetic characterisation in relation to the objectives of the IronFEMME Study by the Laboratory of Paediatrics of the University of Cantabria (Santander, Spain), following the protocols of confidentiality and clinical safety, ensuring the anonymity of the samples and their use for research only.

Five millilitres of whole blood from each patient were collected in EDTA and sent to the Laboratory of Paediatrics of the University of Cantabria. DNA was extracted from each sample using the QIAamp^®^ DNA Blood Mini Kit from QIAGEN (Hilden, Germany), and these samples were preserved at −20 °C. Genotyping for each SNP was performed using real-time PCR assays with TaqMan^®^ SNP genotyping (Applied Biosystems, Foster City, CA, USA).

### 2.13. Statistical Analysis

The statistical analysis will be supervised by a biostatistician and the final analysis will be decided depending the data types and the objectives. The statistical analysis was conducted using the software package SPSS for Windows, version 25.0 (IBM Corp., Armonk, NY, USA). Analysis of normality was confirmed with the Shapiro-Wilk test or the Kolmogorov-Smirnov test (according to sample size). If data were not normally distributed, these were analysed with a non-parametric Friedman ANOVA test to assess differences among phases and time of measurement. The Wilcoxon signed rank test was conducted to test differences between OC phases. When data were normally distributed, two-way repeated measures ANOVA or the linear mixed model for repeated measures were performed to analyse phase, time of measurement and phase*time interaction effects on the main variables of the study. Where appropriate, the Bonferroni post-hoc test was applied to examine pairwise comparisons of each significant factor. Statistical significance was set at *p* < 0.05.

## 3. Discussion

Research on female training responses and exercise adaptations is poor compared to the body of research in males [91]. The fact, including women in research has been seen as a barrier due to the complexity of studying hormone fluctuations associated with the menstrual cycle [92]. However, these hormonal changes should be considered when the exercise response is evaluated on the basis of the increasing participation of women in sports and training programs [93].

The IronFEMME Study was designed to examine the influence of the sex hormone environment on iron homeostasis and exercise-induced muscle damage by considering the different reproductive status present throughout the well-trained female lifespan. The strengths of this study should be highlighted: 1. This is the first study analysing these mechanisms in premenopausal females on three moments of the menstrual cycle coinciding with the most pronounced changes in sex hormones (EPF, LFP and MLP) or on the two phases of an oral contraceptive cycle (WP and APP), following an intra-subject design. 2. This study uses a robust methodology to verify menstrual cycle phases consisting of retrospective calendar counting, blood analysis of sex hormones and urine-based tests to predict ovulation. These are accurate mechanisms to monitor the main hormonal environments occurring throughout the menstrual cycle, as it has been recently suggested [36]. 3. This is the first study comparing active postmenopausal females with active premenopausal eumenorrheic females considering the entire menstrual cycle. 4. Phases were randomised and counterbalanced to avoid the learning effect and repeated bout effect. 5. The exercise protocols were similar to training sessions regularly performed by well-trained females. Future research should address some limitations of this project: (1) The exercise protocols selected may not be intense enough to trigger a noteworthy inflammatory response; (2) The variability between subjects is high and this could influence the lack of differences between phases; (3) Hormonal variability could be high for the same woman between different menstrual cycles and even within one menstrual cycle. Sex hormone concentrations could also vary between days within the same oral contraceptive cycle especially during the withdrawal phase. Hence, it would be interesting to perform daily measurements of sex hormones in both groups; (4) Finally, muscle damage has been evaluated through indirect markers which is reliable, but the lack of histological muscle damage evaluation through muscle biopsies could also be considered as a limitation to address in future studies.

Finally, we hope to clarify the influence of sex hormones on some aspects of female physiology. The exercise response was evaluated in the EFP, LFP and MLP using three-step method to verify the menstrual cycle phase: calendar counting, blood analyses confirmation and urine-based ovulation kits. In addition, the exercise protocols were also performed in the APP and WP to evaluate the influence of oral contraception. A group of postmenopausal females was included as well to examine their response and to compare it to the eumenorrheic female response in their different menstrual cycle phases. Therefore, the IronFEMME Study is well-positioned to provide a global perspective of women’s different reproductive profiles over the lifespan, and their influence on iron metabolism and exercise-induced muscle damage. 

## 4. Conclusions

IronFEMME Study is attempting to provide a global perspective of women’s different reproductive profiles over the lifespan, and their influence on iron metabolism and exercise-induced muscle damage.

## Figures and Tables

**Table 1:** Descriptive data of the participants selected for the study (mean ± SD).

	Study I			Study II		
Variables	Eumenorrheic	OC Users	Postmenopausal	Eumenorrheic	OC Users	Postmenopausal
*n*	37	31	16	20	31	16
Age (years)	30.0 ± 6.3	25.1 ± 4.3	51.4 ± 3.7	28.8 ± 6.2	25.1 ± 4.3	51.4 ± 3.7
Body weight (kg)	59.8 ± 15.7	56.2 ± 10.9	56.7 ± 8.3	57.5 ± 13.8	56.2 ± 10.9	56.7 ± 8.3
Height (cm)	163.7 ± 6.3	163.1 ± 5.5	161.7 ± 4.9	163.9 ± 6.4	163.1 ± 5.5	161.7 ± 4.9
Training experience * (years)	7.7 ± 5.1	7.3 ± 5.5	7.9 ± 3.4	6.4 ± 4.1	3.1 ± 1.9	3.1 ± 1.9
Training volume * (h/week)	5.5 ± 0.9	3.4 ± 1.5	4.1 ± 1.2	7.5 ± 2.1	2.5 ± 1.4	1.6 ± 0.9

* Study I: endurance training experience and volume; Study II: resistance training experience and volume. OC: oral contraceptive.

## Data Availability

Not applicable.

## Abbreviations

1RM	One repetition maximum
APP	Active pill phase
CMJ	Counter movement jump test
CRP	C reactive protein
DOMS	Perceived delayed onset muscle soreness
DXA	Dual-energy X-ray
EFP	Early-follicular phase
EIMD	Exercise-induced muscle damage
FSH	Follicle stimulating hormone
HR	Heart rate
IL-6	Interleukin-6
IronFEMME	Iron and muscular damage: FEmale Metabolism and Menstrual cycle during Exercise
ISAK	International Society for the Advancement of Kinanthropometry
LDH	Lactate dehydrogenase
LFP	Late-follicular phase
Mb	Myoglobin
MLP	Mid-luteal phase
OC	Oral contraceptive
RER	Respiratory exchange ratio
ROM	Range of movement
SNPs	Genetic single nucleotide polymorphisms
STRAW	Stages of Reproductive Aging Workshop
TNF-α	Tumor necrosis factor α
TSH	Thyroid-stimulating hormone
VAS	Visual analogue scale
VCO2	Carbon dioxide production
VE	Pulmonary ventilation
VO2	Oxygen uptake
VO2peak	Peak oxygen uptake
vVO2peak	Maximal aerobic speed
WP	Withdrawal phase
